# A Patient Panel Assignment Strategy to Balance Provider Workload in Family Medicine

**DOI:** 10.3390/healthcare14142178

**Published:** 2026-07-19

**Authors:** Yu-Li Huang, David R. Rushlow

**Affiliations:** 1 Robert D. and Patricia E. Kern Center for the Science of Health Care Delivery, Mayo Clinic, Rochester, MN 55905, USA; 2 Department of Family Medicine, Mayo Clinic, Rochester, MN 55905, USA; rushlow.david@mayo.edu

**Keywords:** family medicine, primary care, patient assignment, panel size, workload balance

## Abstract

**Background/Objectives**: Appointment volume is a primary measure of provider workload and a key contributor to provider burnout in primary care settings. It is highly driven by the size and patient mix of patient panels assigned to providers. To ensure that workload is balanced among providers, an effective patient panel assignment strategy is essential. **Methods**: A patient panel assignment framework was developed and demonstrated using a year of data from a large family medicine practice to assign patients evenly among providers with the following steps: 1. Predicting patient appointment needs using patient characteristics and Adjusted Clinical Group (ACG) scores; 2. Placing patients in appointment groups based on predictive appointment needs; 3. Determining the patient cap of each appointment group for each provider group; 4. Allocating patients from each appointment group to each provider group without exceeding the patient cap. **Results**: The results were demonstrated across 100 simulated reassignments to a retrospective dataset. The variations in the number of appointments among providers within a provider group were compared between current practice and the proposed framework. For the largest provider group with 14 providers, the proposed framework with appointment prediction using all features reduced the standard deviation and range by 85% and 84%, respectively. While the proposed framework improved appointment workload balance among providers, 11.7% of patients required reassignment. **Conclusions**: The proposed framework demonstrates its utility in supporting workload balance. Future work is needed to minimize patient reassignments while accounting for patient care continuity and to incorporate other contributing factors into the framework.

## 1. Introduction

In current primary care settings, provider burnout has reached critical levels, significantly impacting both healthcare quality and workforce satisfaction [1,2]. The primary reasons for burnout include increased patient care demand, workforce shortages, and high administrative burdens [3,4,5]. As patients with long-term conditions are primarily managed in primary care settings [6], appointment volume and patient mix are central to this increased provider workload [7,8]. In the US, patients are assigned a primary care provider (PCP) at the first visit in a facility for better care management [9], and each PCP has their own panel of patients [10]. Although patients can be seen by other providers due to PCP availability at the time of the appointment request, panel size dictates the provider’s workload [11]. When patient panels are poorly balanced, some providers face disproportionate administrative and clinical burdens, leading to exhaustion, while others may remain underutilized [12,13]. Traditionally, patient panel management has relied on simple headcounts, assigning an equal number of patients to each provider regardless of individual patient needs. However, this method fails to account for the patient mix and panel management time differences in appointment needs [14]. A panel of 1500 relatively healthy young adults requires vastly different resources than a panel of 1500 elderly patients with multiple chronic comorbidities. Studies conclude that care team composition can mitigate workload, thereby reducing burnout [15] and increasing patient care continuity [16]. Nonetheless, workload imbalance at the PCP panel level is believed to be the fundamental issue [17,18]. To achieve true workload equality, an effective assignment strategy must move beyond panel size and incorporate the predicted intensity of care required by each patient.

Workload balancing has been studied from emotional and psychological aspects [19,20,21] but requires tools such as questionnaires for evidence. Other studies are mainly based on patient visits and acuity [22,23]. While existing literature has explored factors affecting primary care providers’ workload and burnout [24,25,26,27], and models for predicting workload [28,29], there remains a significant gap in translating these predictive metrics into systematic assignment frameworks that can be applied in dynamic clinical environments. Specifically, there is a need for models that integrate clinical indicators, such as the Adjusted Clinical Group (ACG) score for patient risks [30], with demographic characteristics to forecast appointments accurately [31,32,33]. Most primary care systems are built around the concept of a primary care provider who manages care for patients and their family members, unlike in Asian countries [34]. Hence, family medicine management not only needs to determine the patient panel size for each primary care provider [35,36] but also needs to account for patient complexity and care continuity [37]. This study aims to develop a patient panel assignment framework designed to distribute workload evenly among providers. Utilizing a year of longitudinal data from a Family Medicine department in US, we propose a four-step methodology that predicts appointment needs, categorizes patients into groups, and establishes provider-specific caps to guide assignment and reassignment. This study primarily presents the use of the framework to simulate the reassignment effectiveness of workload balancing among providers. By comparing it with current practice, we demonstrate its potential to reduce workload variance, thereby enhancing operational efficiency.

## 2. Methods

Mayo Clinic Family Medicine Department in Rochester, MN, USA had about 105 paneled providers (43 male and 62 female) from 1 January 2019 to 31 December 2019 with a total of 82,881 completed patient visits. The data was extracted from EPIC Clarity. Providers were categorized into different groups based on their commitment to patient care, depending on their roles and panel management time (PMT). PMT is the time dedicated to patient care, and it ranges from 0.2 to 0.8, which is the percentage in relation to a 1.0 full-time equivalent. PMT includes both visit care and non-visit care. Visit care considers patient contact time for both face-to-face and virtual visits. Non-visit care time includes chart review, visit documentation, planning for patients’ subsequent management, handling patient messages, and so on. Roles can be classified into MD, MD-DD, APP, and resident. MD is a physician type, and MD-DD is another physician type with an additional commitment to be the doctor of the day (DD), providing consultation to medical staff and overseeing clinical operations. APPs are advanced practice providers, such as nurse practitioners and physician assistants. The practice currently does not consider patient service needs when assigning patients to a provider panel. For the same group of providers, panel sizes should be similar, but the number of appointments generated from each panel varies, which causes an imbalanced workload. Hence, a method that can identify patients’ appointment needs to more evenly assign or reassign patients to each provider panel is paramount.

**Figure 1:**
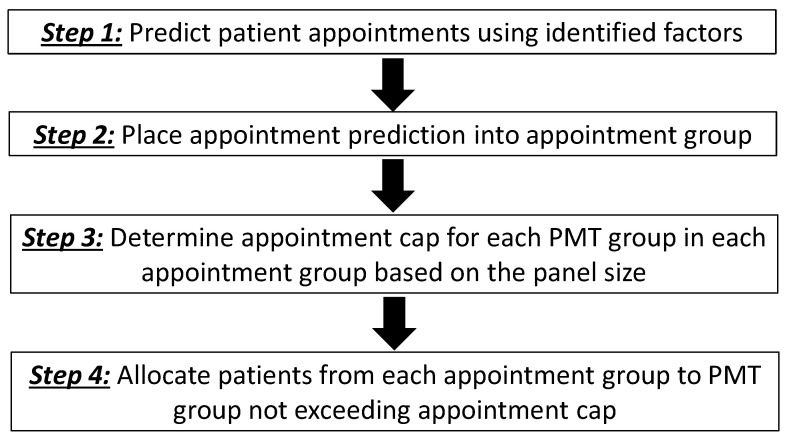
Patient assignment steps.

To balance workload among family medicine providers in terms of appointments for face-to-face and virtual visits, at the time of planning for the following year, we proposed a 4-step framework described in Figure 1. The patient allocation step applies to assigning new patients to a provider panel and reassigning patients from one provider panel to another. Family medicine providers’ panel patients and their appointment data was used to demonstrate the framework step by step. The patient allocation step applies to assigning new patients to a provider panel and reassigning patients from one provider panel to another. The results of this framework focused on reassigning since they were demonstrated through retrospective data.

### 2.1. Step 1

The first step was to predict how many appointments a patient could generate given known factors. The factors included in this study are listed in Table 1. Table 1 describes each factor’s level of detail, including the distribution, average and standard deviation of appointments. ACG is a relative measure of an individual’s expected need for health services. G1 has an ACG of zero, G2 is between 0 and 0.5, G3 is between 0.5 and 1.0, G4 is between 1.0 and 2.0, and G5 is 2.0 and above. Patient characteristics, such as age, gender, geographic location, race, ethnicity, and language, were collected. The patient’s primary insurance carrier was also believed to be a contributor. Other factors, such as whether a patient was a portal user and required an interpreter for medical services, were also included. The portal is an online communication system that allows patients to see medical records and test results, manage appointments, message medical staff, and view payments. These factors or features were available in our system and were used to predict the number of appointments. There are many methods that can be used to predict the number of appointments. In this study, we presented the two most inherently interpretable prediction methods: a regression model [38] and a decision tree method [39]. In addition, we used a feature stratification method with all factors listed in Table 1 or with ACG only. This method clustered patient populations in detail based on the combination of each level from each factor.

### 2.2. Step 2

**Figure 2:**
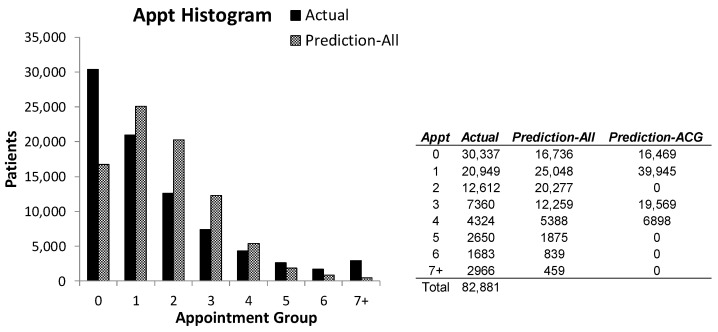
Histograms and summaries of the actual and the prediction using all feature combinations and ACG only for appointment groups.

Given the levels defined in each factor, there existed a total of 2970 combinations. Each combination had its own average number of appointments, which was used to predict appointments. For example, consider a 40-year-old non-Hispanic white male patient who had an ACG of G2, lived in town, spoke English, was an active portal user and had Mayo insurance. This population generated approximately one appointment per year. The distribution of the number of appointments for 2019 is shown in Figure 2. The actual appointments showed an exponential declining trend, and the maximum number of appointments a patient generated was approximately 40. Eight appointment groups were defined: 0 was the patient group that had no appointments, and 7+ was the group that had at least seven appointments. Figure 2 also shows the predictions by all features and ACG only in step 1.

### 2.3. Step 3

After placing patients in their predicted appointment groups, we then decided how to distribute patients evenly among each provider group by first defining the patient cap. Each provider group was defined by provider role and panel management time (PMT). There were four provider roles: medical doctor (MD), medical doctor with doctor of the day (MD-DD), advanced practice practitioner (APP), and resident. Panel management time is a fraction of the time providers spend on patient visits, i.e., 0.5 means providers spend 50% of their time seeing patients. The parameters are denoted as follows:

*i* = provider group index = 1, 2, …, 20

*j* = appointment group index = 0, 1, 2, 3, 4, 5, 6, and 7+

*n_i_* = the number of providers in provider group *i*

*s_i_* = the patient panel size per provider in provider group *i*

*T_i_* = = *n_i_* × *s_i_* is the total number of panel patients in provider group *i*

*T* = total number of panel patients = ∑ *T_i_*

*p_i_* = = *T_i_*/*T* is the percentage of panel patients in provider group *i* relative to total panel patients

*A_j_* = the number of predicted panel patients for appointment group *j*. They were determined in step 2. For the prediction using all features described in step 1, *A*_0_ = 16,736, *A*_1_ = 25,048, *A*_2_ = 20,277, *A*_3_ = 12,259, *A*_4_ = 5388, *A*_5_ = 1875, *A*_6_ = 839, and *A*_7+_ = 459.

*c_ij_* = *A_j_* × *p_i_* is the cap for the panel patient distribution of appointment group *j* to provider group *i*. For example, for the MD role with PMT of 0.5, given *n*_3_ = 14 and *s*_3_ = 1301, *T*_3_ = *n*_3_ × *s*_3_ = 14 × 1301 = 18,218. *T* was calculated to be 82,881. The *p*_3_ = *T*_3_/*T* = 18,218/82,881 = 22.0%. Hence, given an appointment group of one appointment per year (*A*_1_ = 25,048), the number of patients distributed to this group is *c*_31_ = *A*_1_ × *p*_3_ = 25,048 × 22.0% = 5506, as shown in Table 2.

### 2.4. Step 4

Once the patient cap for each provider group in each appointment group was set, the next step was to assign patients from each appointment group randomly to each provider group. For instance, the cap for the MD role with a PMT of 0.5 (*i* = 3) in one appointment group (*j* = 1) was calculated to be 5506. Among the total of 25,048 patients in one appointment group, we randomly selected 5506 patients and assigned them to this provider group. These 5506 patients were then distributed randomly and evenly among the 14 providers, which was approximately 393 patients per provider. The random assignment could also be driven by patient and provider preferences as long as they did not exceed the cap. Patient preferences could be based on provider selection and having the same provider for all family members. It should be noted that reassignment only occurred when existing patients exceeded the cap in the appointment group.

## 3. Results

### 3.1. Appointment Prediction Method Results

The two feature stratification methods, using all feature combinations and ACG only, as well as the two inherently interpretable predictive models, a linear regression and a decision tree, were presented. A fivefold cross-validation with an 80/20 split was used to create the training and testing datasets. Within each fold, 80% of the data was used for training and 20% for validation. Table 3a summarizes the prediction accuracy in comparison with actual appointment groups from the consolidation of the testing datasets from each fold. The overall accuracy was around 40%, with the ACG-only model being the highest. However, ACG predicted five appointment groups since there were only five levels defined and failed to identify patients with a higher number of appointment needs. The prediction using all feature combinations produced more consistent accuracy across appointment groups and had higher predictability for the 7+ group. In addition, we reported the mean absolute error (MAE) for each prediction method (see Table 3b). The all-features model generally has fewer prediction errors, besides appointment groups of 3 and 4. Thus, the model using all feature combinations was selected, and the prediction results of this model on the consolidated testing dataset were used for the remaining steps.

### 3.2. Patient Allocation Method Results

**Figure 3:**
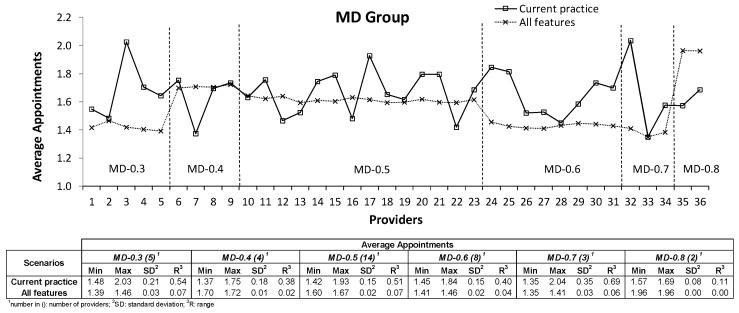
Scenario comparison in average appointments for each provider and summary in MD group.

In 2019, the participating department did not use any factors to predict patient appointments when assigning patients to provider panels. However, the correlation between panel size (number of patients assigned to a panel) and the associated appointments was 0.85, which is relatively high. To ensure a fair comparison, we measured the average number of appointments, calculated as the total appointments divided by panel size. Two scenarios were compared: (1) current practice, and (2) patient assignment using the all features prediction model. The results are demonstrated for the MD group, as shown in Figure 3. For example, there were 14 providers (providers 10 to 23) in the PMT of 0.5 group (MD-0.5). Since they were in the same group, they should presumably have a similar workload in the average number of appointments per patient with the same panel size. In current practice, provider 17, who had the highest number of appointments, was 36% higher than provider 22, who had the lowest number of appointments. Figure 3 also summarizes the two scenarios in terms of the minimum, maximum, standard deviation, and range of average appointments per patient among all providers in this group across 100 simulated reassignments. The simulation assumed that patients could be reassigned across different provider groups. Patients who were reassigned would randomly select a provider whose panel needed them to reach the cap. The standard deviation and range represent the variability of average appointments among providers. The range is the difference between the maximum and minimum values. The 95% confidence intervals were also reported and calculated as average appointments per patient ± 1.96 × standard deviation from these 100 reassignments.

For standard deviation, current practice had a variation of 0.15 in average appointments among providers. The proposed allocation method, mainly reassignment, using the all-features prediction model reduced this variation by 85% [(0.15 − 0.02)/0.15]. In terms of range, the reduction was 84% [(0.51 − 0.08)/0.51]. Figure 3 also shows that the average appointment variation and range in the current state for each of the six provider groups were much higher than those produced by the proposed framework, since they were outside the 95% confidence interval. In addition, current practice introduced inconsistency in panel sizes within each provider group, which was due to manual panel size adjustments over time and contributed to workload imbalance. The panel sizes ranged from 515 to 1503 patients in the group with a PMT of 0.3 (MD-0.3), from 926 to 1380 patients in the group with a PMT of 0.4 (MD-0.4), from 954 to 1633 patients in the group with a PMT of 0.5 (MD-0.5), from 946 to 1650 patients in the group with a PMT of 0.6 (MD-0.6), from 1069 to 1520 patients in the group with a PMT of 0.7 (MD-0.7), and from 1622 to 1711 patients in the group with a PMT of 0.8 (MD-0.8). Step 3 of the proposed framework determined the optimal panel size for each of these groups to remove this inconsistency (see *s_i_* in Table 2). The framework supported appointment workload balance with 11.7% patient reassignments among providers in the same group.

## 4. Discussion

**Figure 4:**
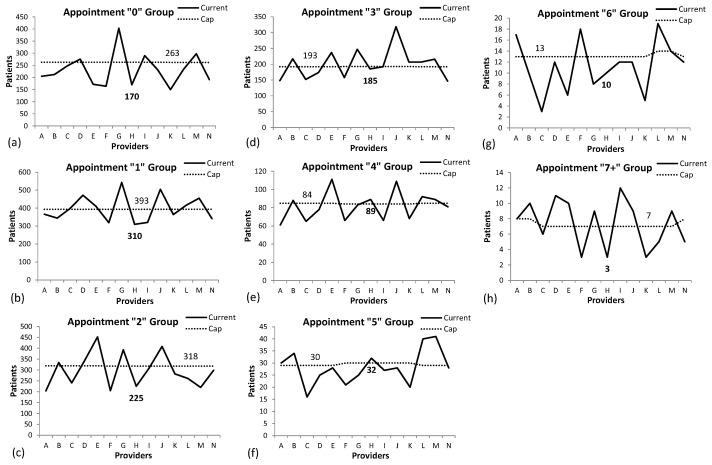
A visual display of patient assignment for MD PMT 0.5 provider group at each appointment group (**a**–**h**).

We presented a framework that can assist in balancing provider workload in a family medicine setting. The purpose of the prediction model is not to perfect individual appointment predictions but to achieve an approximate stratification of patients into workload-relevant groups for aggregate panel balancing. One of the key assumptions is that patients can be reassigned to other providers when their conditions change and require more medical attention. In practice, this assumption is possible but not preferred. Therefore, one way to keep track of providers’ appointments is to develop a systematic view for each provider group. This view should display the number of patients currently predicted in each appointment group (see Figure 4a–h). For the MD provider group with a PMT of 0.5, the proposed framework using the all-features prediction model determines the cap for appointment “1” group to be 393 patients per provider. Providers D, G, J, and M exceed this cap. Thus, if there is a new appointment “1” patient who needs to be assigned, the assignment should go to providers who are currently below the cap. Another way of viewing this display is to examine the difference, combining all appointment groups for each provider, from current assignment to the cap, calculating the difference in percentage. For instance, Figure 4 shows that the patient caps decided for each appointment group from 0 to 7+ are 263, 393, 318, 193, 84, 30, 13, and 7 patients. Figure 4 also labels the actual number of patients assigned to each appointment group as 170, 310, 225, 185, 89, 32, 10, and 3 for provider H. The percent difference for appointment group “0” is (170 − 263)/263 = −38%. Hence, the percent differences from the cap are −38%, −19%, −36%, −18%, −21%, −30%, 38%, and −57%. We then calculate the expected appointment difference (sum of the percent differences multiplied by the expected appointments) for provider H. The calculation is (−38%) × 0 + (−19%) × 1 + (−36%) × 2 + (−18%) × 3 + (−21%) × 4 + (−30%) × 5 + 38% × 6 + (−57%) × 7 = −5.7, which means provider H is 5.7 appointments per patient on average below the cap. The ideal expected appointment difference per patient should be close to zero.

### 4.1. Impact of Prediction Error to Patient Panel Reassignment

The results from Table 3 indicated that the prediction accuracy was around 40% and much lower for the higher appointment groups. The anticipated reassignment should have shown some improvement in workload balance but not as much as expected. To explore the actual impact of the prediction errors, we investigated one assignment for a provider in the MD group with a PMT of 0.5; see the appointment distribution in Table 4. This provider should have had approximately 2100 appointments with a panel size of around 1301 patients. The shaded numbers were the accurate prediction. The reassignment overestimated 475 patients (unshaded portion) and underestimated 264 patients (light-shaded portion) in the left panel of Table 4. The right panel in Table 4 indicates the corresponding appointments from the left panel. However, the under- and overestimation seem to compensate for each other and result in an annual total of 2074 appointments for this provider. In addition, we found that the average deviation from target appointments was around 30 appointments. It is certain that the performance could be much improved with higher prediction accuracy, especially for higher appointment groups. A future study is needed to discover other methods and develop prediction uncertainty metrics for tracking [40].

### 4.2. Limitation and Future Work

This study focuses on balancing workload among providers in terms of the number of appointments, assuming patients can be reassigned to a different provider panel. Although the framework shows promising results, the following limitations cannot be overlooked:

- There are other workload measures beyond visit frequency, such as administrative complexity, documentation demands, psychosocial burden, team dynamics, and patient acuity. In this study, the administrative burden was incorporated into PMT. Providers with a PMT of 0.5 means only 50% of providers’ time is dedicated to clinical activities. The retrospective dataset in this study embedded practice patterns of 6.5 h as patient contact hours. The rest of 1.5 h is used for charting. Psychosocial burden [41], team dynamics for patient safety [42], and patient acuity measures besides ACG score should also be considered when predicting provider workload.
- Both variable panel size and patient reassignment in the same PMT group are difficult concepts in current primary care practice. This work focuses on patient reassignment to balance provider workload among appointment groups while maintaining the same panel size among providers in a PMT group. The average of 11.7% patient reassignment consists of 20%, 7%, 14.8%, 6.8%, 9.4%, 12.2%, 25.3%, and 17.5% for appointment groups 0, 1, 2, 3, 4, 5, 6, and 7+, respectively. Reassigning patients in appointment group 0 may not have a care continuity issue since patients are generally healthier with fewer medical needs. However, for appointment groups 6 and 7+, a high percentage of reassignments is not preferred. Studies have revealed the importance of care continuity, leading to clinical responsibility, physician knowledge, and patient trust as critical elements for patient well-being and satisfaction [43,44]. A mitigation strategy could be reassigning these patients among providers in the same care team, which is a potential future study. In addition, an optimization approach could be a potential solution to minimize reassignment, especially for higher appointment groups, while keeping panel sizes as close as possible.
- Approximately 60% of the providers in this study were female. Studies have reported that female providers have more responsibilities outside of work, including childcare and elder care, leading to dissatisfaction with work–life integration [45]. The proposed framework in its current form did not account for provider gender difference in calculating optimal panel patients in each appointment group. It is essential to take provider differences into account when assigning patients for fair clinical workload.
- The framework was demonstrated in a large family medicine practice setting. The evidence of applicability to others is limited, especially for smaller clinics, resource-limited systems, non-US healthcare structures systems, and organizations without an ACG infrastructure. The implementation challenges remain unknown even for the participating family medicine practice, as many studies have identified challenges in system limitations and performance realignments [46].
- The study selected the feature-stratification method for patient appointment prediction. However, this method requires an exhaustive search for all feature combinations, which can be extremely sparse for some. In our dataset, 41% out of 2970 combinations had fewer than five patients, making their representation questionable for accurate appointment prediction. The results indicated that the overall accuracy was affected by class imbalance, especially for the higher appointment groups. A further study is needed to lessen the impact of the sparseness on workload balance and on other prediction methods to improve accuracy.

Lastly, the unanswered question is whether the concept of patient panel reassignment is acceptable to primary care providers. To the best of our knowledge, there is very limited literature on this topic. One study reported that panel patient adjustments occur only due to a heavy disease load and that the provider needs to be acknowledged [47]. This will be the biggest hurdle for implementing such a framework. The framework considers all patients regardless of new patient assignment or existing patient reassignment. Additional considerations such as appointment length for different patient types [48] should be included in the allocation method. Equitable workload balancing should not be based only on appointment volume and panel size. Future models should incorporate non-visit workload, team support, documentation burden, patient complexity beyond ACG, and provider-specific constraints.

## 5. Conclusions

The findings of this study underscore the critical role of data-driven patient panel management in mitigating appointment workload imbalance among primary care providers. By transitioning from traditional panel sizing to a predictive framework based on patient characteristics and Adjusted Clinical Group (ACG) scores, healthcare administrators can achieve a more equitable distribution of patient assignments. While the framework necessitates a level of patient reassignment that may impact established relationships, the gain in workload stability offers a viable path toward sustainable practice environments. It is worth noting that prospective validation is needed before operational implementation. Future refinements should focus on integrating more contributing factors into appointment prediction and care team structures to protect care continuity while optimizing the allocation process to minimize patient reassignment disruption.

## Figures and Tables

**Table 1:** Factors available for appointment prediction.

	Patients	Appointments			Patients	Appointments	
Factors	Count	Average	SD ^1^	Factors	Count	Average	SD ^1^
*ACG* ^2^				*Insurance*			
G1	16,469 (20%)	0.0	0.0	Commercial	24,733 (30%)	1.3	1.9
G2	39,945 (48%)	1.3	1.3	Mayo	40,542 (49%)	1.7	2.0
G3	9934 (12%)	2.6	2.2	Medicaid/MiscGovt	7910 (10%)	1.8	2.5
G4	9635 (12%)	2.9	2.3	Medicare	3535 (4%)	2.6	3.0
G5	6898 (8%)	4.2	3.8	MedicareAdvantage	3086 (4%)	2.8	3.0
*Age*				Self-Pay/Unknown	1926 (2%)	1.0	2.1
1–25	25,692 (31%)	1.6	2.0	WorkersCompensation	1149 (1%)	2.4	2.6
26–30	8237 (10%)	1.2	1.9	*Race*			
31–65	40,645 (49%)	1.5	2.1	Asian/Other	7735 (9%)	1.3	1.9
66–80	6418 (8%)	2.5	2.6	Black/PacificIslander	2578 (3%)	1.5	2.1
80+	1460 (2%)	3.6	3.7	White/Native	72,568 (88%)	1.7	2.2
New	429 (1%)	3.3	1.9	*Language*			
*Gender*				English	79,962 (96%)	1.7	2.2
F	45,355 (55%)	1.8	2.3	Other	2919 (4%)	1.3	2.0
M	37,526 (45%)	1.4	2.0	*Interpreter*			
*Location*				N	81,644 (99%)	1.6	2.2
Close	80,113 (97%)	1.7	2.2	Y	1237 (1%)	1.6	2.2
In State	477 (1%)	1.3	2.0	*Ethnicity*			
Out of State	2291 (3%)	0.8	1.6	HispanicLatino	1316 (2%)	1.6	2.2
*Portal*				NotHispanicLatino	81,565 (98%)	1.6	2.2
N	26,871 (32%)	1.6	2.1				
Y	56,010 (68%)	1.7	2.2				

^1^ SD: Standard Deviation. ^2^ ACG: Adjusted Clinical Group.

**Table 2:** Summary of cap determination for each provider and appointment group.

			Provider (*n_i_*)	Panel Size (*s_i_*)	Panel Patients (*T_i_*)		Patients in Appointment Group *j* (*A_j_*)							
*i*	Role	PMT				% (*p_i_*)	0 (16,736)	1 (25,048)	2 (20,277)	3 (12,259)	4 (5388)	5 (1875)	6 (839)	7+ (459)
1	MD ^1^	0.3	5	874	4372	5.3%	883	1321	1070	647	284	99	44	24
2		0.4	4	1159	4636	5.6%	936	1401	1134	686	301	105	47	26
3		0.5	14	1301	18,218	22.0%	3679	5506	4457	2695	1184	412	184	101
4		0.6	8	1320	10,561	12.7%	2133	3192	2584	1562	687	239	107	58
5		0.7	3	1248	3743	4.5%	756	1131	916	554	243	85	38	21
6		0.8	2	1571	3142	3.8%	634	949	769	465	204	71	32	17
7	MD ^1^–DD ^2^	0.3	1	1004	1004	1.2%	203	303	246	148	65	23	10	6
8		0.4	2	956	1912	2.3%	386	578	468	283	124	43	19	11
9		0.5	2	1074	2149	2.6%	434	649	526	318	140	49	22	12
10		0.6	1	1169	1169	1.4%	236	353	286	173	76	26	12	6
11	APP ^3^	0.2	1	207	207	0.2%	42	63	51	31	13	5	2	1
12		0.3	2	384	767	0.9%	155	232	188	114	50	17	8	4
13		0.4	5	244	1220	1.5%	246	369	298	180	79	28	12	7
14		0.5	6	778	4669	5.6%	943	1411	1142	691	304	106	47	26
15		0.6	13	515	6694	8.1%	1352	2023	1638	990	435	151	68	37
16		0.7	4	683	2733	3.3%	552	826	669	404	178	62	28	15
17		0.8	5	656	3281	4.0%	663	992	803	485	213	74	33	18
18	Resident	0.2	1	445	445	0.5%	90	135	109	66	29	10	5	2
19		0.3	20	425	8507	10.3%	1718	2571	2081	1258	553	192	86	47
20		0.4	6	575	3452	4.2%	697	1043	845	511	224	78	35	19
Total Panel Patients (*T*)					82,881									

^1^ MD: Doctor or Medicine; ^2^ DD: Doctor of the Day; ^3^ APP: Advanced Practice Provider.

**Table 3:** Model comparison with actual appointment groups for (**a**) prediction accuracy and (**b**) mean absolute error.

(a)									
*Percent Accuracy* ^1^	Actual Appointment Groups								Overall Accuracy
Methods	0	1	2	3	4	5	6	7+	
All Features	54.4%	51.2%	30.0%	30.6%	11.0%	5.8%	3.5%	3.9%	41.2%
ACG Only	54.3%	74.8%	2.9%	39.5%	17.5%	0.0%	0.0%	0.0%	43.6%
Linear Regression	50.0%	51.8%	23.7%	36.5%	16.4%	3.7%	0.1%	0.1%	39.2%
Decision Tree	54.4%	51.5%	30.7%	31.3%	10.5%	6.5%	4.1%	1.7%	41.3%
(**b**)									
***Mean Absolute Error ^2^***	**Actual Appointment Groups**								
**Methods**	**0**	**1**	**2**	**3**	**4**	**5**	**6**	**7+**	
All Features	0.71	0.70	0.79	1.08	1.53	2.16	2.82	5.27	
ACG Only	0.79	0.80	0.82	0.86	1.40	2.23	3.06	5.94	
Linear Regression	0.84	0.76	0.82	1.01	1.48	2.19	3.02	5.84	
Decision Tree	0.74	0.74	0.79	1.09	1.61	2.28	3.01	5.70	

^1^ *Percent Accuracy* calculates the predicted visits over actual visits in an appointment group. ^2^ *Mean Absolute Error* calculates the absolute errors between predicted and actual values in an appointment group.

**Table 4:** Reassignment appointment distribution in relation to actual appointment.

Patients	Actual								Appointments	Actual							
Predicted	0	1	2	3	4	5	6	More	Predicted	0	1	2	3	4	5	6	More
0	263								0								
1	117	171	63	25	10	4	4		1		171	126	75	40	20	24	
2	72	120	70	28	14	7	4	4	2		120	140	84	56	35	24	29
3	15	31	48	39	27	14	10	9	3		31	96	117	108	70	60	71
4	10	14	13	14	11	9	3	11	4		14	26	42	44	45	18	86
5		2	4	4	4	6	3	7	5		2	8	12	16	30	18	57
6					2	2	1	8	6					8	10	6	81
More				1	1	1		4	More				3	4	5		42

## Data Availability

The data are available from the corresponding author upon reasonable request due to restrictions related to patient confidentiality and institutional data protection policies.

## Abbreviations

The following abbreviations are used in this manuscript:

ACG	Adjusted Clinical Group
PCP	Primary care provider
PMT	Panel management time
DD	Doctor of the day
APP	Advanced practice provider
MD	Medical doctor
